# Overview and update on cytomegalovirus-associated anterior uveitis and glaucoma

**DOI:** 10.3389/fpubh.2023.1117412

**Published:** 2023-03-01

**Authors:** Zifan Ye, Yumei Yang, Weishaer Ke, Yuhang Li, Kaijun Wang, Min Chen

**Affiliations:** ^1^Eye Center of the Second Affiliated Hospital, School of Medicine, Zhejiang University, Hangzhou, China; ^2^Zhejiang Provincial Key Lab of Ophthalmology, Hangzhou, China; ^3^Shangyu People's Hospital of Shaoxing, Shaoxing, China

**Keywords:** cytomegalovirus, anterior uveitis, glaucoma, intraocular pressure management, antiviral therapy

## Abstract

Cytomegalovirus anterior uveitis is the most common ocular inflammatory disease caused by cytomegalovirus infection. It mainly occurs in middle-aged males with competent immunologic function, and the incidence is higher in Asia. The clinical manifestations vary from Posner-Schlossman syndrome and corneal endotheliitis to Fuchs uveitis syndrome, and are often accompanied by intraocular hypertension. Secondary glaucoma is a potentially blinding ocular complication with a pathogenesis that includes complicated immunological factors, intraocular inflammation, different types of angle abnormalities, and the administration of steroids, which may result in physical discomfort and visual impairment. Diagnostic tests, such as the polymerase chain reaction, optical coherence tomography, ocular microscopy, and confocal microscopy, might help in identifying anterior uveitis caused by other viruses. Combinations of antiviral medications and anti-inflammatory agents are effective treatments. If pharmacological therapy cannot reduce intraocular pressure or slow the progression of glaucomatous optic neuropathy, surgical intervention is required as a last resort.

## 1. Introduction

Cytomegalovirus (CMV), a large double-stranded DNA virus of the herpesvirus family, is transmitted perinatally, by close contact, including sexual contact or parenterally. CMV can cause anterior uveitis (AU) and even secondary glaucoma in the immunocompetent population. A previous study showed that 52.2% of eyes with presumed Posner-Schlossman syndrome (PSS) and 41.7% of eyes manifesting symptoms similar to Fuchs uveitis syndrome (FUS) were CMV positive, as determined by the polymerase chain reaction (PCR) (1). CMV-associated AU is mainly characterized by elevated intraocular pressure (IOP), corneal endothelial damage, and the presence of varying degrees of keratic precipitates (KPs). At present, the pathogenesis remains unclear. The prevalence of CMV-associated uveitis exhibits marked geographic differences, attributable to factors such as economy, population density, health care status, life style, and environmental exposure in various regions (2). Most cases have been reported from Asia, especially from China and Japan, which could be resulted from the higher seroprevalence of CMV in Asia (3). (The percentage of CMV-associated AU in different regions of the world is presented in the Supplementary Table 1).

Ganciclovir and its derivatives can competitively inhibit DNA polymerase, thereby inhibiting DNA synthesis and CMV replication. However, the high recurrence rate of CMV-associated AU often confuses clinicians regarding treatment, although a low dosage of persistent antiviral drugs may help to reduce recurrence. One issue that has not been completely resolved involves the risks and benefits of antiviral therapy, as the antiviral agents may present their own risks of systemic effects, such as granulocytopenia, thrombocytopenia, anemia, and elevated serum creatinine levels (4). While most cases of CMV-associated AU can be managed medically or by surgery, early identification, comprehensive cognition and effective treatment are required as it may reduce loss of visual function and health-related life quality, as well as the social and economic burdens.

The aim of this review was to summarize recent work on CMV-associated AU, including the characteristics, pathogenesis, diagnosis, and current management of this disease, to provide a reference for clinical treatment.

## 2. Characteristics

### 2.1. Posner-Schlossman syndrome

Posner-Schlossman syndrome (PSS), also known as glaucomatocyclitic crisis, is an ocular condition characterized by recurrent acute attacks of mild, unilateral, non-granulomatous AU with severely elevated IOP (5). During the acute attack, the typical hallmarks are corneal edema with KPs, open anterior chamber angle, and normal visual fields and optic discs (5). The symptoms include blurring vision, with or without ocular discomfort or pain, lasting for a few hours to several weeks, and resolving spontaneously (6). IOP appears to be normal during the interictal periods (7). The disease is commonly benign; however, 25% of cases can develop glaucomatous optic atrophy if the attacks happen frequently enough or if the duration is sufficiently long (8).

Infections are contributing factors for PSS, with CMV as the most likely culprit. Early in 1987, Bloch-Michel et al. first reported the detection of CMV antibodies in the aqueous humor of PSS patients (9). Chee et al. have since carried out a series of studies on the relationship between CMV infection and PSS in recent years. They analyzed the aqueous humor of 105 immunocompetent uveitis patients *via* the polymerase chain reaction (PCR) and found that 18 of 24 patients whose eyes tested CMV-DNA-positive were ultimately diagnosed with PSS. Moreover, anti-CMV medications had a definite effect on the treatment of these patients (10). In other research, active CMV infection in the anterior chamber was detected in 35 eyes of 67 patients diagnosed with PSS (1). Moreover, an association was reported between the recurrence of PSS and CMV infection, and the amount of CMV DNA was positively correlated with IOP (11).

### 2.2. Corneal endotheliitis

Corneal endotheliitis is a unique clinical entity that is manifested by corneal edema, KPs, and mild anterior chamber inflammation and leads to varying degrees of corneal endothelial dysfunction or increased IOP (12).

CMV has increasingly been recognized as one of the most common infection factors of corneal endotheliitis. Infection may occur after intraocular or corneal surgery, and most commonly after corneal transplantation. At the time of diagnosis, 97.1% of patients had a medication history of topical glucocorticoid use, and 25.7% of patients had a history of corneal transplantation, which may require postsurgical use of regular glucocorticoids or immunosuppressants (13). Therefore, decreased ocular immune function may be an alternative etiology of this disease. However, CMV-associated corneal endotheliitis typically occurs in immunocompetent individuals. Koizumi et al. first reported the presence of CMV in the aqueous humor among immunocompetent patients with corneal endotheliitis (14).

The symptoms of corneal endotheliitis include photophobia and visual disturbances, usually occurring in a single eye. The physical findings can be variform KPs, with or without overlying cornea edema, ocular hypertension, or pigmentation changes in the trabecular meshwork (12). Coin-shaped KPs are most common, observed in 70.6% of eyes, whereas the linear pattern occurred in 8.3% of eyes, according to a Japanese series (13). The presence of coin-shaped KPs is reportedly a positive predictor for CMV infection (90.9% positive predictive value) (15). As with PSS, CMV corneal endotheliitis may involve the presence of concomitant intermittent elevation of IOP, most of which can be adequately controlled after the anti-CMV treatment (16).

### 2.3. Fuchs uveitis syndrome

Fuchs uveitis syndrome is a chronic, typically unilateral (90%) disorder, predominantly manifesting as heterochromia, asymptomatic mild inflammation involving the anterior uvea and vitreous, cataract, and secondary glaucoma (17). It was initially described by Fuchs in 1906, and despite the proposal of many hypotheses at that time, the infectious theory remained the most probable. FUS likely represents a mild form of immunoreactivity operating in the final common infectious pathway (18). Rubella virus is the main causative agent of FUS in Europe (90–100%) and the USA (19), while CMV is the predominant cause in Asia (20). Chee et al. reported that 41.7% of eyes with presumed FUS were CMV positive and that the patients were more likely to be male, with advanced age at diagnosis, and with nodular endothelial lesions (1).

FUS does not present acute symptoms of severe pain, photophobia, or ocular redness (21). The major clinical signs are characteristic KPs, anterior chamber cells and flare, and vitreous opacities, suggesting the presence of low-grade inflammatory reactivity (21, 22). Heterochromia is not a ubiquitous sign of FUS; however, iris stromal atrophy, which frequently results in heterochromia, is always present (22). The visual changes associated with FUS are frequently secondary to other complications, such as cataract and glaucoma (22, 23). The prevalence of secondary glaucoma is approximately 12.8–27.6% in FUS patients (21, 24). Normal range levels can be achieved by cataract extractions of eyes with complicated cataracts. Secondary glaucoma is the most common cause of permanent visual loss and remains an intractable aspect to control therapeutically (21).

## 3. Pathogenesis

### 3.1. Immunological factors, latency, and reactivation of CMV

Complex immune regulation is involved in CMV-associated AU. The most important immune responses against CMV are CD4^+^ and CD8^+^ T cells. The CD8^+^ T cell response mainly focuses on a small number of viral proteins, such as immediate-early protein IE-1 and tegument phosphoprotein pp65 (16). Miyazaki et al. confirmed that CD8^+^ T cells could be directly stimulated by CMV-infected corneal endothelial cells and that viral peptide pp65 could induce the release of interferon-γ (IFN-γ) (25). When the initial infection has been controlled, the highly activated effector T cells, which secrete cytokines like IFN-γ and kill virus-infected cells, initiate the apoptosis process. The long-term control of CMV in immunocompetent hosts requires considerable effort, and one hallmark is the maintenance of large populations of CMV-specific memory CD8^+^ T cells, which differentiate rapidly upon encountering CMV again (26). IE-1 transcripts have been detected with murine CMV up to 3 months post viral injection, and the related epitope is rapidly expressed on the reactivation of latent virus. Consequently, IE-1–specific T cells may be associated with immune surveillance and the prevention of full reactivation (27).

The mechanisms regulating the latency and reactivation of CMV remain ambiguous. Clinical observations indicate that corneal endothelial lesions always start from the periphery and spread to the center of the cornea, implying that the surrounding tissues, such as the trabecular meshwork (TM) or ciliary body, may serve as reservoirs of CMV (28). The *in vitro* experiment of Choi et al. revealed homogeneous masses of electron-dense material and viral particles in the cytoplasm of TM cells and determined that human TM cells could effectively support CMV replication (29). A subsequent histopathological study on cadaveric eyes showed that the CMV inclusion bodies are mainly localized in the iris, ciliary body, and endothelial cells of the Schlemm's canal (30). These findings provide experimental support for viral colonization in the physiological outflow facility.

Apart from intraocular reservoirs, CMV can be detected in the spleen, mesenteric lymph nodes, and salivary glands after intraocular infection. Zinkernagel et al. found comparable kinetics responses in secondary lymphoid organs to those elicited by intraocular infection in a murine model (27). This can probably be explained by the dissemination of CMV *via* the Schlemm canal and uveoscleral outflow, as well as the rapid spread to the secondary lymphoid organs.

Inflammation may result from local CMV reactivation in the anterior segment or could be generated by macrophages and monocytes. Tissue macrophages and their potential progenitors, the monocytes, serve as latent cell reservoirs and reactivation sites and are established target cells of CMV (31). Reactivation of lytic viral replication in co-cultures of macrophages and mouse embryo fibroblasts indicates that macrophages can serve as reservoirs for CMV incubation (32). Macrophages recognize CMV particles and form IFN I within hours post-infection to limit viral spread (33). In the early phase, macrophages induce NK cell recruitment and IFN-γ production by inflammasome activation (31). Monocytes are heterogeneous immune factors that participate in both the innate and adaptive immune responses and can be divided into classical, intermediate, and non-classical subsets (34). Classical monocytes have inflammatory properties, whereas non-classical monocytes have patrolling properties, and intermediate subsets can present antigens (34). Classical inflammatory monocytes infected by CMV recirculate into the bone marrow and differentiate into patrolling monocytes, which carry the virus and have a long half-life. Viral replication can be reinitiated when monocytes are activated and differentiate into macrophages (35). Inflammation and cytokines, such as tumor necrosis factor (TNF) and IFN-γ, are important factors in the process of reactivation (36).

### 3.2. Mechanism of intraocular pressure elevation

A highly complex relationship exists among the factors that determine IOP, including fluctuating aqueous production and drainage, open or closed angle, and responses to steroids. The mechanisms of IOP elevation may vary throughout the course of the disease, with the open-angle mechanism considered dominant. The obstruction of the TM by inflammatory cells, proteins, debris, fibrin, or inflammatory precipitates may partly explain the initially elevated IOP. Concurrently, substances suspended in the aqueous humor can increase viscosity (37). Moreover, inflammatory edema occurring in the TM can block the outflow channel of the aqueous humor in the early stage of infection. Animal experiments have shown swelling of the trabecular lamellae and reduction in the intertrabecular spaces might increase the outflow resistance and lead to increased IOP (38). Thereafter, permanent scarring of the trabecular meshwork and the Schlemm canal, as well as the formation of the vascular membrane in the anterior chamber angle, can block the flow of aqueous humor and lead to a permanent IOP increase that is sometimes unresponsive to medical therapy (39, 40).

The effect of steroids might also result in an open-angle mechanism of elevated IOP. Steroids are thought to increase aqueous secretion and reduce aqueous outflow, thereby leading to morphological and biochemical changes in the TM, including the accumulation of glycosaminoglycans, the reduction of phagocytic activity by the trabecular endothelial cells, alterations in the TM cell size, and changes in the cytoskeletal organization (41). Approximately one-third of patients with uveitis are high-steroid responders and can develop elevated IOP (42). This increases the difficulty of distinguishing between the original inflammation and the side effects of steroids (42). Topical steroid testing may assist in identifying unilateral uveitic glaucoma (43).

Secondary angle closure can result from closure of the synechiae, appositional closure, or neovascularization of the angle after a long duration of open-angle uveitis or secondary glaucoma (37). The accumulation and organization of inflammatory debris and cells in the angle give rise to peripheral anterior synechiae (PAS), which pull the iris forward. Even a limited extent of PAS can lead to a compromised outflow facility (41). Insufficient or delayed treatment results in the formation of adhesions between the lens capsule and the iris surface, or between the iris and cornea, with pupil blockage as a consequence (40). Moreover, inflammatory cytokines, on rare occasions, can enhance anterior neovascularization and lead to fibrovascular closure and neovascular glaucoma (37).

Inflammatory cytokines and chemokines may also have a considerable function in the regulation of outflow with CMV infection. Choi et al. demonstrated an enhancement of transforming growth factor-β1 (TGF-β1) in CMV-infected TM cells in the early phase (29). TGF-β1 is an upstream cytokine that regulates the outflow facility by inducing characteristic structural changes in TM cells. The administration of ganciclovir in the early stage of infection has no effect on the production of TGF-β1; robust elevation of the inflammatory cytokines, monocyte chemotactic protein-1 (MCP-1) and interleukin-8 (IL-8) is subsequently induced (44). MCP-1, a member of the CC subfamily of chemokines, effectively mediates the influx of monocytes and macrophages into inflammatory lesions, while IL-8 is a potent chemoattractant for neutrophils (45). The stimulation of MCP-1 and IL-8 increases the formation of actin stress fibers, focal adhesions, and the contraction of TM cells, thereby potentially regulating the outflow and IOP (44).

## 4. Investigation

### 4.1. Anterior chamber tap and viral detection

#### 4.1.1. Polymerase chain reaction

For uveitis patients with highly suspected CMV infection, virus detection should be performed as soon as possible. Viral serology is not helpful in establishing a definite diagnosis, because most adults have had previous exposure to CMV. Consequently, PCR of CMV DNA in the aqueous humor is commonly used throughout the world. An aqueous tap should be done during the IOP spike, preferably before the initial therapy (46). PCR detection has high sensitivity and specificity (5), and microdrip digital PCR exhibits better sensitivity than quantitative PCR (47). A negative result cannot exclude CMV infection, as a positive result may be obtained after repeated aqueous taps (3).

#### 4.1.2. Goldmann-Witmer coefficient analysis

The Goldmann–Witmer coefficient (GW coefficient) helps to determine pathogen-specific intraocular antibody production. IgM antibodies, which usually appear along with CMV DNA, are produced in the early stage of infection and persist for a short interval. Anti-CMV IgG antibodies are produced at ~1 week after infection and have a lifetime persistence. The CMV DNA-based detection rate (37.5–52.2%) (1, 10) is often lower than the CMV IgG-based detection rate (64–80%) (9, 11). The rate of serum CMV IgG antibody in the normal population can reach a level of up to 87% (48). When the blood–eye barrier is damaged, antibodies and other serum protein components leak into the eyes, leading to false-positive results. The GW coefficient is calculated as follows: (anti-CMV IgG in aqueous humor/total IgG in aqueous humor)/(anti-CMV IgG in serum/total IgG in serum) (49). It is used to obtain a preliminary determination of whether ocular-specific antibodies occur *in situ* or are a result of blood–water barrier leakage, thereby excluding false-positive results. A GW coefficient <2.0 indicates no *in situ* antibody production, whereas a GW coefficient ≥ 4.0 indicates robust *in situ* antibody production in the eye. However, the GW coefficient is not in wide global use, and PCR seems to be the sole choice relied on by most countries (50).

#### 4.1.3. Metagenomic next-generation sequencing

Metagenomic next-generation sequencing (mNGS) is a new technique developing recently, and compromises high-throughput sequencing and bioinformatics that detect pathogens (51). This test can detect fungi, parasites, bacteria, and viruses in intraocular fluid samples for ocular infections (52). mNGS performs unbiased sequencing of the genome in the sample, thus it is able to detect the commensal and opportunistic microbes simultaneously with the potential pathogen. As a high-throughput sequencing technology, it requires more costs and time, compared to quantitative PCR. Previous studies that used mNGS for detecting pathogens like CMV and HHV-6 revealed that metagenomic DNA sequencing was highly consistent with pathogen directed PCRs, which is promising for diagnosis of CMV-associated AU (52).

### 4.2. *In vivo* evaluation

*In vivo* evaluation includes confocal microscopy, specular microscopy, and anterior segment optical coherence tomography (OCT). These offer a quick check compared to PCR and GW coefficient analysis and provide a more intuitive demonstration of anterior segment lesions, especially in the cornea.

Corneal *in vivo* confocal microscopy (IVCM) is a non-invasive technique that provides corneal detail in each layer at the cellular level (53). It has been used to detect and monitor both pathologic and infectious conditions (54). The “owl eye” morphology, considered a clue to CMV infections, presents as large corneal endothelial cells with an area of high reflection in the nuclear region, surrounded by a ring structure of low reflection (55). Confocal microscopy can detect the “owl eye cells” in patients with CMV corneal endotheliitis. This sign is well-correlated with the coin-shaped lesions observed by slit-lamp microscopy and may represent necrotic endothelial cells (55).

Specular microscopy reveals decreases in corneal endothelial cell density. Miyanaga et al. reported a significant association between the endothelial cell loss intensity and CMV viral load in the aqueous humor (56). Some of the endothelial cells present spots that resemble “owl eye” (57).

The anterior segment OCT also represents an effective non-invasive alternative for diagnosing and monitoring CMV-associated AU. Protruding structures of diverse shapes have been observed at the posterior cornea (58). High reflectivity of the endothelial cell layer and deep stromal cornea can also be distinguished (58). Due to the improvement of corneal edema, these high-intensity regions can be resolved after antiviral therapy.

## 5. Treatment

### 5.1. Pharmacological treatment

#### 5.1.1. Antiviral treatment

To date, no clear and consolidated guidelines are available for the administration of drugs for the treatment of CMV-associated AU. Human CMV responds well to ganciclovir and valganciclovir, which are available in oral, intravitreous, or topical application forms (59). The antiviral effect of ganciclovir requires intracellular phosphorylation with the assistance of a cellular kinase, UL97 viral kinase, and UL54 DNA polymerase. Through stepwise phosphorylation, ganciclovir is converted to ganciclovir triphosphate, which remains stable in CMV-infected cells for several days (60). As a prodrug, valganciclovir hydrolyzes to ganciclovir before producing antiviral effects. Notably, antiviral treatment generally suppresses viral replication rather than eradicating latent viral DNA from infected cells (61). Thus, a lifetime risk of subsequent recurrences remains, and the response to antiviral therapy can be delayed according to individual variations (Different methods of administration for antiviral therapy are listed in the Supplementary Table 2).

##### 5.1.1.1. Systemic administration

Once CMV infection is confirmed, systemic antiviral therapy should be initiated immediately to inhibit viral replication. Ganciclovir, the first anti-CMV drug, has been used clinically since 1984. To achieve an effective tissue concentration, ganciclovir is administered intravenously at an induction dose of 5 mg/kg two or three times daily for adult patients with normal renal function (59, 62). Oral valganciclovir is presently a common option, for the absolute bioavailability generated from valganciclovir, at 60% (63), is much higher than that of ganciclovir, which is only 6% to 9% (64), and its use eliminates the inconvenience of daily injections. The effective systemic regimen for CMV-associated AU includes oral valganciclovir at 900 mg twice per day for 2–6 weeks as an induction treatment, followed by a maintenance treatment of 450 mg twice per day for a minimum of 4 weeks (62, 65).

As an induction treatment, a dose of 900 mg valganciclovir orally administered twice a day is as effective as the administration of intravenous ganciclovir twice daily at a body weight of 5 mg/kg (66). This regimen has also been confirmed by Touhami et al., who showed that both induction regimens seemed similar in terms of initial response, best-corrected visual acuity (BCVA) changes, and reduction in the number of attacks per year in patients with CMV-associated AU (62).

The timing and duration of antiviral therapy have important effects on prognosis. Compared to patients who received delayed antiviral therapy, the requirement for surgery was lower in patients whose antiviral treatment was initiated early (62). The duration of maintenance indicates control for up to 6 months by inhibiting viral DNA replication while limiting the possible emergence of CMV-resistant strains and the potential risk of side effects (67). Ganciclovir and its related metabolites are excreted *via* glomerular filtration and tubular secretion; therefore, patients with impaired renal function usually have a prolonged half-life and a higher plasma concentration. Myelosuppression is another adverse effect, usually manifested as neutropenia, thrombocytopenia, and leukopenia, and prompts a dosage decrease, thereby favoring viral resistance (68). Consequently, the dosage needs adjustment in patients with renal insufficiency, and periodic laboratory monitoring should be undertaken routinely during medication (69).

##### 5.1.1.2. Intravitreal injection

Animal models have shown that the vitreal concentration of ganciclovir after intravitreal injection is ~100 times higher than the concentration after intravenous injection (70). This makes intravitreal injection more cost-effective while also decreasing the side effects of systemic administration.

Overall, clinical studies involving intravitreal ganciclovir treatment have revealed inconsistent outcomes. Hwang and associates favored intravitreal ganciclovir as a loading dosage, with or without adjunctive oral valganciclovir, according to the severity of the post-injection inflammation index (67). A single intravitreal injection of ganciclovir (2 mg/0.05 mL) as a loading dose or multiple injections at a dose of 2 mg/0.1 mL weekly for 3 months were the recommended strategies (71). All their patients responded to the intravitreal therapy, and the long-term outcome was a median 50% recurrence-free survival time of 47.0 months. However, in a comparative study, Chee et al. suggested that intravitreal administration of ganciclovir might not be as effective as systemic treatments in terms of inflammation control. They found that intravitreal injection had a lower response (57%) and a higher relapse rate (100%) compared with systemic administration (the response rate was 89%, while the relapse rate was 82%) and ganciclovir implant (the response rate was 100%, while the relapse rate was 100%) (71). The mean aqueous drug level after systemic administration of ganciclovir is ~3.2 mg/L, whereas the concentration is 0.23 mg/L at 72 h, and 0.006 mg/L at 7 d after a single intravitreal injection of 2 mg ganciclovir, which does not even reach the 50% inhibitory dose (ID_50_) for CMV (0.25–1.22 mg/L) (71). However, infection and retinal tears or detachment remain threats during intravitreal injection procedures.

##### 5.1.1.3. Local eye medication

Topical ganciclovir is an alternative antiviral therapy for CMV-associated AU. Commonly used local eye medications include ganciclovir implants, 0.15% ganciclovir gels, and 2% ganciclovir eye drops.

Intraocular implants containing ganciclovir (Vitrasert; Bausch & Lomb Inc., Claremont, California, USA) are very popular in the industrialized world (72). These implants contain 4.5 mg of ganciclovir in a non-biodegradable drug delivery system and provide an intraocular level of ganciclovir five times higher than that achievable with systemic administration. The implant is also effective for up to 8 months, and systemic toxicity is greatly reduced (72). This treatment was initially aimed at treating CMV retinitis, with implantation into the vitreous. The implant could also be sutured at the site of the pars plana, thereby offering another tentative option for the treatment of anterior segment CMV infection (71). Chee et al. reported that ganciclovir implants had a good response rate in CMV-associated AU and endotheliitis eyes, but the recurrence rate was high after the period of validity, and an equally high recurrence rate also appeared in the second course of treatment (71). Consequently, although initially approved by the FDA, this implant has been discontinued.

Ganciclovir gel is reported to have a moderate response rate, but the recurrence rate is also lower than with other regimens. The recommended application of 0.15% ganciclovir ophthalmic gel is often 6–8 times per day for induction and 1–4 times per day for maintenance (3). To some extent, the usage mode may reduce patient compliance. In a study using high-performance liquid chromatography, Chee et al. found that local eye medication with ganciclovir could not achieve an effective drug concentration in the aqueous humor (10). The same group also found that the drug levels in the aqueous humor were again below the ID_50_ for inhibition of CMV replication following the application of 0.15% ganciclovir gel, and were weakly correlated with the central corneal thickness (CCT) (73). Despite these theoretical flaws, CMV was eradicated from the aqueous humor after a 6-week application of 0.15% ganciclovir gel in all patients who strictly followed the medication instructions (73).

Su et al. reported the efficacy of custom-made 2% ganciclovir eye drops in 68 CMV-positive PSS patients. All 68 eyes (100%) exhibited positive outcomes, including remission of anterior chamber inflammation, reduction of corneal edema, and good IOP control after 1 month. CMV was eliminated in all aqueous humor samples after 3 months. The recurrence rate was 11.6% at the end of the average follow-up period of 39.8 months, which was much lower than the recurrence rate of 57.14% previously observed using 0.15% ganciclovir gel (71, 74).

##### 5.1.1.4. Combined treatment

Ganciclovir works by suppressing viral replication rather than having a virucidal effect; consequently, a need remains for additional long-term antiviral treatment. The current literature suggests that antiviral treatment should be continued for at least 3 months (74, 75). However, to counteract the side effects and poor compliance associated with long-term medication, combined treatments, such as oral valganciclovir with intravitreal injection or topical ganciclovir, can be employed. A loading dose is also recommended, followed by long-term treatment to increase efficacy and reduce recurrence (76).

One study showed that a combination of intravitreal injections and systemic antiviral therapy appeared to have good outcomes in a 15-month follow-up, as none of the eyes had recurrent intraocular inflammation (67). However, subsequent extension of the observation period revealed recurrences, with a 50% recurrence-free survival of nearly 4 years for this therapeutic regimen (77). Sobolewska et al. combined 0.15% ganciclovir gel with oral valganciclovir in both the induction and maintenance periods and found that 64% of the patients could stop therapy without relapse after an average of 14 months of treatment (75). Harada and coworkers reported 100% responsivity for a duration as long as 6 months an antiviral treatment regimen that combined oral valganciclovir with topical ganciclovir eye drops, and 58.3% of their patients successfully terminated treatment (78). Some studies have suggested that the use of systemic or topical treatments alone may produce a higher percentage of recurrence compared with combined treatments (79). A multicenter study compared the efficacy of systemic, topical, and combination treatments in 106 patients, and the effective rate was better for the combination treatment (63.7%) than for topical or systemic administration alone (52.3%) (13). Thus, different administration protocols can be used simultaneously in serious cases.

#### 5.1.2. Anti-inflammation treatment

Most of the visible damage to the eye arises from the immunoreaction secondary to the viral infection, rather than from the infection itself. Therefore, anti-inflammation medication is necessary as an adjuvant therapy in cases of CMV-associated AU with stromal or endothelial keratitis. When treatment is needed, the goal is complete suppression of inflammation (80). Corticosteroids, which inhibit the cyclooxygenase pathways of the inflammatory response, are often the preferred choice. Topical drops are the easiest and most commonly used method of administration and have the least side effects. The final drug selection is based on the severity of the anterior uveitis. Potent steroids, such as prednisolone acetate, should be administered in cases showing severe inflammatory reactions, whereas mild AU cases can be treated with weak topical steroids, such as betamethasone or dexamethasone.

If the inflammation becomes advanced, bilateral, or recurrent after a single topical administration, or if any component of posterior uveitis is evident, then systemic or periocular routes should be considered (81). The initial treatment should be a high dose of oral steroids during the acute phase, and this should then be tapered in successively smaller decrements to decrease the risk of relapse (82). The medication process needs to continue throughout the disease. The variable duration of the effect after relapse is a problem with periocular injections. Despite the recovery after reinjection, each relapse leads to cumulative damage, resulting in poor visual outcomes termed the “saw-tooth decline” (80).

For individuals who are recalcitrant to steroids, weak steroids or steroids with the least propensity for raising the IOP (e.g., 1% rimexolone) are common substitutions. In addition, topical non-steroidal anti-inflammatory drugs, such as flubriprofen or diclofenac sodium, may be other options (71).

Notably, not every case of anterior uveitis requires anti-inflammatory treatment. Little benefit is gained by administering steroids in Fuchs uveitis syndrome (80). Ophthalmologists should pay close attention to the side effects such as cataract and glaucoma secondary to the long-term use of steroid therapy.

#### 5.1.3. Intraocular pressure lowing therapy

IOP control is vital in the management of CMV-associated AU and secondary glaucoma to protect the optic nerve from damage. Traditionally, medications that reduce aqueous production (including beta blockers and carbonic anhydrase inhibitors) are considered as first-line therapies, assuming the patient has no systemic contraindications. The absorption by topical routes may be reduced in cases with corneal edema; therefore, systemic carbonic anhydrase inhibitors are effective agents when topical routes fail to control the ideal IOP (83). Intravenous or oral hyperosmotic agents, such as mannitol and glycerol, can also be used as temporizing measures in acute episodes of ocular hypertension.

Some controversy exists regarding the use of prostaglandin analogs (PGAs) in CMV-associated AU. These agents are thought to lower IOP by enhancing uveoscleral outflow. Nevertheless, the theoretically higher risk of anterior uveitis, blood-aqueous barrier disruption, and cystoid macular edema has made ophthalmologists reluctant to use PGAs as a first-line therapy (37). However, controlled clinical trials have not provided strong evidence in support of an association between PGAs and the recurrence of inflammation or cystoid macular edema (84). Another concern is related to the potential for viral reactivation, as a risk of recurrence has been proven for herpetic keratitis and CMV-associated AU in immunocompetent individuals (85). Prostaglandin synthetase inhibitors are helpful in reducing the recurrence of epithelial herpes simplex virus (86); therefore, PGAs may participate in the final pathway involved in stimulating viral recurrence. Therefore, PGAs should be used with caution in eyes with previous episodes of CMV infection.

Pilocarpine and anticholinergic agents are generally contraindicated in the treatment of uveitic glaucoma due to the potential exacerbation of inflammation related to blood–aqueous barrier breakdown (42). These agents can induce miosis, further shallowing the anterior chamber, decreasing the uveoscleral outflow, and possibly predisposing the patient to posterior synechiae and pupillary membrane formation (41).

### 5.2. Surgical procedures

Generally, the prognosis of CMV-associated AU is good with timely treatment with antiviral therapy, steroids, and pressure-lowering agents. Nevertheless, the outcomes can be complicated by cataract formation, corneal decompensation, and glaucoma. According to previous reports, glaucoma develops in 23% of eyes with acute CMV-associated AU and in 36% of eyes with chronic CMV-associated AU (1, 61, 87). Overall, 20–60% of the patients can be unresponsive to antiviral therapy (39, 62, 75). In these cases, anti-glaucoma surgery should be considered to help stabilize the IOP and prevent vision loss.

The first-line surgery is minimally invasive glaucoma surgery (MIGS), including Trabectome^®^ handpiece or iStent inject^®^ implantation (88). Opening the Schlemm's canal from the inside can be less traumatic. Filtration surgery can supplement as a second-line surgery, including the implantation of an Ahmed valve and trabeculectomy with or without mitomycin C (89). Cyclophotocoagulation and cryotherapy may be considered as substitutions when all other approaches fail (90).

MIGS and trabeculectomy can both reliably reduce IOP and the medication burden (91), with similar failure rates (MIGS: 37.7% vs. trabeculectomy: 25–51.9%) (88). The reduction effect is maintained for a shorter time after MIGS than after trabeculectomy (88). Zhong et al. stated that trabeculectomy was an effective method for decreasing uncontrolled IOP (50 ± 5 mmHg) to a normal range (14 ± 2 mmHg) for over 2 years without any anti-glaucoma medications (91). By comparison, anti-glaucoma medications (a decrease from 3.1 ± 0.4 to 0.8 ± 1.1 mmHg) are still required for 12 months after Trabectome^®^ surgery for a notable reduction in IOP (a decrease from 40 ± 10 to 13 ± 1 mmHg) (92). Similar conclusions were made by Pohlmann et al., who found that the IOP reduction effect started to fail in 56% (9 out of 16) of MIGS cases within 2 years (88). By contrast, by attempting to increase the physiological outflow facility, the preservation of the ocular surface makes Trabectome^®^ surgery a valuable option for CMV-associated AU. The greatest advantage of MIGS is its favorable safety profiles: postoperative side effects, such as sustained hypotony, choroidal effusion or hemorrhage, infection, or a BCVA loss > *second* line therapy due to glaucoma, are seldom reported (90). According to the statistics, IOP spikes are the most frequent MIGS-associated complications (90).

## 6. Conclusion

CMV-associated AU is a complicated entity. The clinical manifestations vary differently between individuals with PSS, corneal endotheliitis or Fuchs uveitis syndrome. There is growing recognition that its pathogenesis is related to the complex immune and inflammatory process caused by viral infection. However, the association between CMV genotypes and various responses in different target cells in the anterior segment, and the exact mechanism underlying IOP elevation need further investigation.

This review summarized examinations commonly used in clinical diagnosis, and current management in CMV-associated AU treatment. Indeed, antiviral therapy is the major strategy. Due to the latency and recurrence of CMV, so far there is still a lack of evidence-based consensus guideline on antiviral treatment. Corticosteroids and intraocular pressure lowering therapy can be added as assistances. However, as a double-edged sword, corticosteroids should only be applied after initiation of antiviral therapy, as it can promote viral replication. Various glaucoma drainage surgeries have an increasing role in the management of CMV-associated uveitic glaucoma. MIGS, cyclophotocoagulation and cryotherapy offer new alternatives on the basis of traditional filtration surgery, and MIGS has shown success in surgical safety. Larger well-designed studies are required to determine the long-term efficacy of those procedures.

## Author contributions

ZY and YY wrote the first draft. WK and YL made contributions to the retrieval of literature. MC and KW made revisions of the manuscript. All authors contributed to the critical revision and provided final approval of the submitted version of this article.
