# Supplementary material for: Overview and update on cytomegalovirus-associated anterior uveitis and glaucoma
Source: Front Public Health. 2023 Mar 1;11:1117412. doi: 10.3389/fpubh.2023.1117412 (PMC10014866; doi:10.3389/fpubh.2023.1117412)
Supplement: Supplementary file 1 [file Table_1.DOCX]

Supplementary Material

**Supplementary Table 1.** The percentage of cytomegalovirus-associated anterior uveitis in different regions of the world.

| **Author, Year** | **Country** | **Number of uveitis patients** | **AU/uveitis patients (%)** | **CMV-associated AU/AU patients (%)** |
| --- | --- | --- | --- | --- |
| De-la-Torre et al, 2009 | Colombia | 693 | 200/693 (28.9%) | 1/200 (0.5%) |
| Tan et al, 2013 | Singapore | 928 | 552/928 (59.5%) | 13/552 (2.4%) |
| Llorenc et al, 2015 | Spain | 1022 | 534/1022 (52.3%) | 1/534 (0.2%) |
| Sukavatcharin et al, 2016 | Thailand | 758 | 351/758 (46.3%) | 96/758 (12.7%) |
| Chen et al, 2016 | Taiwan, China | 450 | 276/450 (61.3%) | 8/450 (1.8%) |
| Siak et al, 2017 | Singapore | 1249 | 804/1249 (64.3%) | 103/804 (12.8%) |
| Keorochana et al, 2019 | Thailand | 586 | 292/586 (49.9%) | 28/292 (9.6%) |
| Hart et al, 2019 | Australia | 1236 | 919/1236 (74.4%) | 1/919 (0.1%) |

AU: anterior uveitis; CMV: cytomegalovirus.

**Supplementary Table 2.** Different methods of administration for antiviral therapy.

|  | **Author, Year** | **Country** | **Number of cases** | **Therapeutic regimen and medication duration** | **Extra anti-inflammatory agent** | **Follow-up time** | **Outcome** |
| --- | --- | --- | --- | --- | --- | --- | --- |
| **Systematic administration** | Chee et al  2010 | Singapore | 19 | VGCV 900mg po bid × 6 weeks，then 450mg po bid × 6 weeks | 0.12% prednisolone acetate bid | 4.9-54.8 months | failed: 2/19 (10.5%) recurrence: 14/17 (82.4%) |
|  | Touhami et al  2018 | France | 14 | VGCV 900mg po bid × 3 weeks，then 450mg po qid × 4 weeks (at least) | topical steroids and topical and/or oral antiglaucoma medications when necessary | 6.21 months | failed: 0  recurrence: 9/14 (69.2%)  cytolysis: 1/14 (7.1%) |
|  | Touhami et al  2018 | France | 21 | GCV 5mg/kg iv q8h × 10 days，then 450mg po qid × 4 weeks (at least) | topical steroids and topical and/or oral antiglaucoma medications when necessary | 6.13 months | failed: 2 (9.5%)  recurrence: 16 (76.2%)  neutropenia: 1 (4.8%)  anaphylactic: 1 (4.8%) |
|  | Bhoopat et al  2020 | USA | 15 | VGCV 900mg po bid × 2 weeks (at least)，then 450mg po bid after achieving inactive inflammation | none | 48 months | recurrence: 13/15 (86.7%) glaucoma: 8/15 (53.3%) pancytopemia: 1/15 (6.7%) |
| **Local eye medication** | Chee et al  2010 | Singapore | 4 | GCV implant (4.5mg of GCV in drug delivery system) sutured in pars plana × 8 months | 1% prednisolone acetate qid during the immediate postoperative period | 4.9-54.8 months | failed: 0  recurrence: 4/4 (100%) |
|  | Chee et al  2010 | Singapore | 17 | 0.15% GCV gel qid × 3 months (at least) | 0.5% ketorolac tromethamine qid or 0.12% prednisolone acetate bid | 4.9-54.8 months | failed: 6/17 (35.3%) recurrence: 5/11 (45.5%) |
|  | Su et al  2014 | Taiwan, China | 68 | 2% GCV eye drops q3h, then 2% GCV eye drops q4h | topical steroids, and tappered down when anterior chamber infalammation decreased | 40 months | failed: 25/68 (36.8%) recurrence: 5/43 (11.6%) |
|  | Waduthantri et al  2018 | Singapore | 27 | 0.15% GCV gel 5 times/day × 6 weeks | none | 6 weeks | failed: 0 |
| **Intravitreal injection** | Hwang et al  2009 | Taiwan, China | 27 | GCV 2mg/0.05ml intravitreal injection once | none | 12-22 months | failed: 0  recurrence: 0 |
|  | Chee et al  2010 | Singapore | 27 | GCV 2mg/0.1ml qw × 12 weeks | 0.5% ketorolac tromethamine qid or 0.12% prednisolone acetate bid | 4.9-54.8 months | failed: 3/7 (42.9%)  recurrence: 4/4 (100%) |
|  | Cheng et al  2021 | Taiwan, China | 8 | GCV 2mg/0.05ml intravitreal injection once | none | 14.7 months | failed: 0 |
| **Combined treatment** | Hwang et al  2009 | Taiwan, China | 4 | GCV 2mg/0.05ml intravitreal injection once, then VGCV 900mg po bid × 1-4 months | none | 12-22 months | failed: 0  recurrence: 0 |
|  | Sobolewska et al  2014 | Germany | 11 | VGCV 900mg po bid × 3 weeks + 0.15% GCV gel，then VGCV 450mg po bid × 3 months (at least) + 0.15% GCV gel | 1% rimexolone eye drops or loteprednoletabonat eye drops | 10-46 months | failed: 4/11 (36.4%)  recurrence: 2/7 (28.6%) glaucoma: 6/11 (54.5%) |
|  | Harada et al  2018 | Japan | 17 | VGCV 900mg po bid × 3 weeks + 1% GCV eye drops 6 times daily, then VGCV 450mg po bid + 1% GCV eye drops 6 times daily | 0.1% fluorometholone eye drops or 0.1% betamethasone eye drops | 4-67 months | failed:0  recurrence: 6/12 (50%) |
|  | Cheng et al  2021 | Taiwan, China | 53 | GCV 2mg/0.05ml intravitreal injection once, then VGCV 900mg po bid × 1.9 months | none | 14.7 months | failed: 0 |

VGCV: valganciclovir; GCV: ganciclovir; po: per os; iv: intravenous injection; bid: twice a day; qid: four times a day; q8h: every eight hours; q3h: every three hours; q4h: every four hours; qw: once a week.
